# A role for dendroecology in guiding hydrologic restoration and mitigating risks of peatland soil loss: an example from *Chamaecyparis thyoides* forests

**DOI:** 10.1007/s00468-025-02633-x

**Published:** 2025-06-06

**Authors:** Robert B. Atkinson, Abigail Weaver, Joshua A. Kincaid, Frederic C. Wurster, James M. B. Doyle

**Affiliations:** 1 Department of Organismal and Environmental Biology, Christopher Newport University, Newport News, VA 23606 USA; 2 Middle East District, US Army Corps of Engineers, 201 Prince Frederick Drive, Winchester, VA 22602 USA; 3 Great Dismal Swamp National Wildlife Refuge, 3100 Desert Road, Suffolk, VA 23434 USA; 4 Dramby Environmental Consulting, 2707 W. Cary Street STE 03, Richmond, VA 23220 USA

**Keywords:** Historic hydrologic regime, Ditching, Tree rings, Basal area increment, Peatland restoration, Wetlands, Ecosystem Services, Peat fire

## Abstract

**Key message:**

Tree rings of *Chamaecyparis thyoides* exhibit growth responses that are influenced by local hydrologic conditions through decadal timescales. That perspective can assist peatland natural resource managers in selection of hydrologic restoration targets that minimize chronic microbial oxidation and acute peat loss associated with fire.

**Abstract:**

Temperate forested peatlands are valued for myriad ecosystem services including carbon storage and biodiversity which may be lost through anthropogenic disturbance of hydrologic regimes. Hydrologic alterations may be recorded in tree stem growth patterns and provide insights for management and restoration. In *Chamaecyparis thyoides* (Atlantic White Cedar, Juniper) swamps, stand drainage causes a shift from net soil carbon sink to source as microbial oxidation and catastrophic fire oxidize stored organic matter. Here, we analyze historic radial growth patterns in *C. thyoides* in order to characterize drainage history to guide hydrologic management for peat conservation and restoration. Basal area increment (BAI) estimates across a ~ 60-year chronology (1939–2003) were analyzed by flexible beta cluster analysis of 185 trees from 13 *C. thyoides* stands in the Great Dismal Swamp National Wildlife Refuge in Virginia and North Carolina, USA. Stands formed 3 groups, and growth rates among all groups were indicative of a drained hydrologic regime throughout the chronology compared to an undrained control stand. Regime shift analyses identified positive shifts for 2 stand groups in 1954 and for all 3 stand groups in 1963. Multiple response permutation procedures and partial mantel tests both identified two predictive growth variables including (1) visual observations of fluctuation in the water table and (2) proximity to a primary ditch. Growth rate was suppressed when weirs were installed in the mid-1980s; however, growth rebounded within ~ 2 years. The chronology ends when stands were struck by a major hurricane in 2003 and fires in 2008 and 2011 liberated 1.38 Tg of peat carbon. We conclude that dendroecology can detect hydrologic changes through time and can reduce risks of microbial oxidation and catastrophic fire in forested peatlands.

## Introduction

Hydrologic regimes are a master variable in tropical (Dommain et al. 2010) and boreal forested peatlands (Rochefort et al. 2012). In mid-Atlantic peatlands of the temperate region, *Chamaecyparis thyoides* L. (B.S.P.) swamps are continuously saturated (seasonally flooded, saturated hydrologic regime, FGDC 2013) which exerts control over many of the support functions associated with carbon storage. The long-term saturation slows radial growth in individual trees, resulting in tree rings that are narrow, but stand-level primary production is offset by high stem density and by slightly higher litter production, such that primary production rates remain high (Day and Dabel 1978; Day 1979; DeBerry and Atkinson 2014). Most peatland carbon storage occurs in soil both globally (Holden 2005) and in GDS (Sleeter et al. 2017), and persistent soil anoxia and acidity slow peat decomposition (Day 1982; Kalnins 2000; Duttry et al. 2003) and root production (Crawford et al. 2007), due in part to *Sphagnum* dominance in the herb stratum (Shacochis et al. 2003). Thus, when soils are saturated for a long duration, primary production exceeds mineralization resulting in biogenic peat accumulation (Brinson 1993) as described for forested peatlands in boreal regions (Beaulne et al. 2021) and in southeast Asia (Dommain et al. 2010). Fire can be precluded by soil saturation until combustion reaches the canopy, and *C*. *thyoides* stands may persist for centuries (Little 1950; Frost 1998; Zimmermann and Mylecraine 2003). When fire reaches the canopy, sunlight reaches the soil seed refugium and supports dense natural regeneration (Little 1950).

*Chamaecyparis thyoides* is an obligate hydrophyte (USDA 2024) and a coniferous tree that forms near-monocultures in saturated, acidic, non-tidal freshwater swamps, and vast stands were once common in temperate peatlands throughout the mid-Atlantic coastal plain (Dabel and Day 1977; Laderman 1989). Species in the genus *Sphagnum* are drivers in peatland formation in boreal and temperate regions (Rydin and Jeglum 2013), which were present in pollen records during Great Dismal Swamp (GDS) peatland development that followed glacial retreat ~ 10,000 ya (Whitehead 1972; Whitehead and Oaks 1979) and were abundant when forested wetlands replaced herbaceous marshes 3700 ya (Willard et al. 2023). *Chamaecyparis thyoides* stands occur on deep peat soils such as the Pungo Soil Series (Dolman and Buol 1967) where peat and species in the genus *Sphagnum* reduce water table fluctuation, restrict inundation to swales (pools), and maintain saturation to the soil surface above a shallow water table on hummocks via capillary rise (Little 1950; Whitehead and Oaks 1979). The resulting hydrologic regime is classified as seasonally flooded, saturated (FGDC 2013) and *C. thyoides* trees exhibit somewhat complacent, climatically insensitive growth where stands are undrained (Atkinson 2020; Doyle et al. 2021).

With effective ditching, stands shift to temporarily flooded hydrologic regimes, as indicated by wider tree rings and more rapid radial growth which may include greater climatic sensitivity, especially to precipitation and related proxies such as the Palmer Drought Severity Index (Atkinson 2020). Associated changes in carbon-related ecosystem functions include both increased belowground biomass and fine root production (Rodgers et al. 2003), and aboveground changes include faster growth in stem diameter and lower stand stem density (DeBerry and Atkinson 2014). Aeration of the soil reverses the carbon storage role of peat in forested peatlands via rapid microbial oxidation, which has been established both globally (Moore and Dalva 1993; Galloway et al. 1999) and for *C. thyoides* swamps (Akerman 1923; Korstian 1924) including our study sites in GDS (Kalnins 2000; Duttry et al. 2003) as modeled by Sleeter et al. (2017).

Temporarily flooded hydrologic regimes further risk peatland soils via fire. In the temperate region, drained peatland soils are subject to oxidation by fire which converts stands to pocosin (Sharitz and Gibbons 1982; Schafale and Weakley 1990) or open water systems, and carbon losses can be acute, similar to that reported by Turetsky et al. (2011) in northern peatlands and by Dommain et al. (2010) in tropical peatlands. In the temperate region, of the 1.7 Tg combined total of carbon emitted by fires in the Great Dismal Swamp National Wildlife Refuge (GDSNWR) in 2008 and 2011, 1.38 Tg (81%) was from soil (Reddy et al. 2015; Hawbaker et al. 2016; Sleeter et al. 2017).

Historic peatland drainage by ditch and canal construction was intended to support land use activities such as forestry, agriculture, peat harvesting, or grazing (Trettin et al. 1997; Price et al. 2003; Ramchunder et al. 2009), but the effects vary spatially and temporally. The greatest water table drawdown occurs within 30 m of a ditch (Boelter 1970), and drainage may be minimal when stands are greater than 150 m from the ditches (Boelter 1970; Ramchunder et al. 2009). Conversely, during restoration, when ditch drainage is restricted by some form of water control structure, the water table is highest (wettest) adjacent to ditches but remains relatively unchanged farther from ditches. Land use activities can also result in prolonged inundation, as spoil placement or road construction impounds surface water and lengthens hydroperiods, or shortening of hydroperiods downstream of dam construction, which killed hydrophytic tree species in the Amazon (Assahira et al. 2017). *Chamaecyparis thyoides* stands may be killed by inundation or gradually replaced by mesophytic plant species where saturation declines (Seim 2005), but *C. thyoides* persists in some locations and growth rates may record excessive or insufficient moisture in space and time (Atkinson 2020).

Tree growth responses to hydrologic conditions has been reported for many terrestrial ecosystems (Speer 2010) and aquatic ecosystems, including water level dynamics in lakes (Young et al. 1995; Copenheaver et al. 2010); sea-level rise in coastal ecosystems (Kirwan et al. 2007; Bowen 2016; Doyle et al. 2021); stream flow and overbank flooding (Mitsch and Rust 1984; Dudek et al. 1998; Cleaveland 2000; Ford and Brooks 2002; Anderson et al. 2019); water levels of forested floodplains (Keeland et al. 1997; Allen 2016); and hydrologic regimes of bottomland forests (Copenheaver et al. 2007). Hydrologic regime influence on peat condition has been characterized by several authors including in a southeast Asia study (Wosten et al. 2008), annual ring growth patterns among trees in forested peatlands with several tree species in southeast Asia (Dommain et al. 2010), *Picea mariana* in boreal peatlands (Krause and Lemay 2023), *Pinus taeda* in GDS (Phipps et al. 1978), and *C. thyoides* peatland swamps both near their northeastern US range limit (Pearl et al. 2017) and in the mid-Atlantic region where drainage and monthly climate variables are correlated with growth (Merry 2005; Patterson 2011; Patterson and Atkinson 2015; Atkinson 2020; Doyle et al. 2021).

At GDSNWR, a 240-km-ditch network was created beginning in the 1760s and facilitated across landscapes by the Dismal Swamp Canal in 1805. Restoring the hydrology of the swamp to pre-disturbance conditions has been a goal of the U.S. Fish and Wildlife Service since Refuge establishment in 1973 (USFWS 2006; Wurster et al. 2016). While some hydrologic impacts and recovery have been documented (Lichtler and Walker 1979; USFWS 2006; Eggleston et al. 2018; Speiran and Wurster 2021) and *C. thyoides* tree rings have been analyzed in one stand (Atkinson 2020), the timing and extent of drainage and soil saturation throughout the Refuge are unclear, and the impact of drainage history on the extent of two fires and on restoration efforts has not been assessed. The purpose of this study was to determine if radial growth patterns in *C. thyoides* are reliably associated with hydrologic regime in ways that can guide conservation of peat and restoration of *C. thyoides* in mid-Atlantic peatlands.

### Site description

**Fig. 1:**
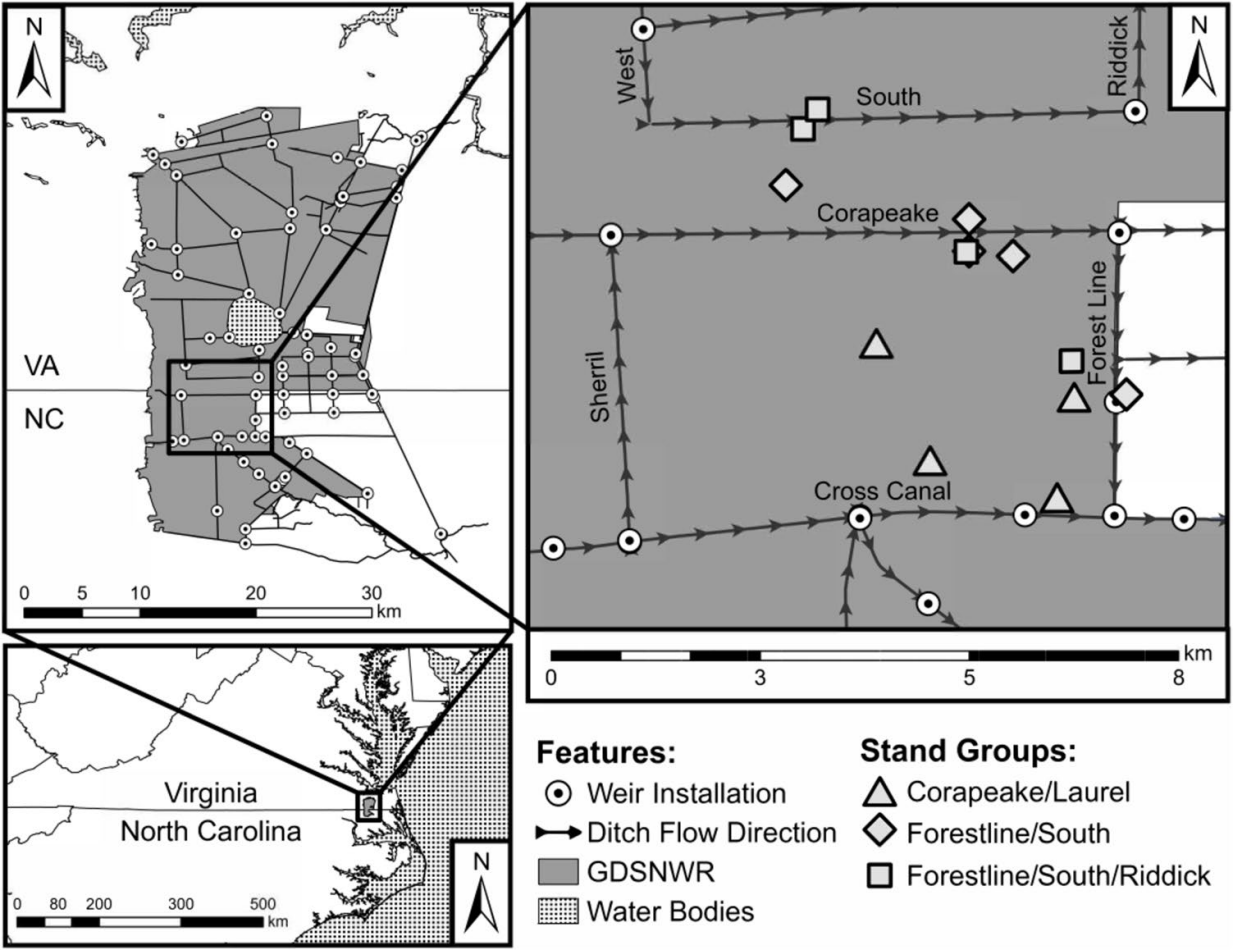
Location of Great Dismal Swamp National Wildlife Refuge (GSDNWR) and an inset map of stands, ditches, and installed weirs. Stand groups are presented in Fig. 2

The > 400,000-ha Dismal Swamp contained the largest stands of *C. thyoides* within its range, which extends from Maine to Mississippi (Laderman 1989). The last 1200 ha of the *C. thyoides* swamp type effectively disappeared from GDS following Hurricane Isabel in 2003, subsequent salvage logging, and two fires in 2008 and 2011. Within that expanse, the GDSNWR, established in 1973, comprises 45,300 ha and is located on the Virginia and North Carolina border approximately 250 km west of the Atlantic Coast (36º32' N, 76º28' W)(Fig. 1). The swamp ranges in elevation from 4.6 to 7.6 m and decreases in elevation approximately 0.2 m/km towards the east (Carter 1987; Laderman 1989). Rainwater is the primary source of water although there are groundwater and surface water contributions from mineral uplands along the western edge of the swamp (Eggleston et al. 2018). Consequently, some areas of the swamp are more fen-like while others are more bog-like, suggesting GDS is a mesotrophic peatland or “poor fen,” intermediate between minerotrophic and ombrotrophic (Mitsch and Gosselink 2007). Soils consist of deep (≥ 2 m thickness) histosols that formed since the most recent glacial retreat (Whitehead and Oaks 1979) and are mostly of the Pungo Soil Series, classified as Typic Medisaprist (Dolman and Buol 1967; Reber et al. 1981). Thompson et al. (2003) sampled one of the GDS stands that is included in this study between 1998 and 2001 and reported soil organic matter content (93%), bulk density (0.16 SE 0.03 g/cc), and pore water pH (3.3–3.6). Woody species reported at this stand included *C. thyoides*, *Acer rubrum* L*.*, *Persea borbonia* (L.) Spreng., *Clethra alnifolia* L., and *Lyonia lucida* C. Koch (Shacochis et al. 2003).

Refuge personnel and others have assembled historical hydrologic information from various sources. Of the 250 km of drainage ditches in the GDSNWR at present, few were constructed prior to 1950. Aerial photographs and older USGS topographic maps suggest that the most intensive ditch construction period occurred between 1950 and 1973 during the last cycle of commercial timber harvests prior to Refuge establishment. Ditches 10 m wide and 2 m deep were constructed by excavating surface peat and underlying fine-to-medium-grained sand, silt, and clay (Eggleston et al. 2018). Road beds were built on top of 10–13 m wide spoil piles adjacent to ditches and rise about 0.3–1.0 m above ambient peat surface. Known ditches in our study area constructed in the 1960s include Corapeake, Forestline, Kim Saunders, Sherill, and South ditches. Cross Canal and Riddick ditches were constructed in the 1800s, but were deepened in the 1950s. The resulting ditch network effectively transported water to lower elevation water bodies including Lake Drummond, the Dismal Swamp Canal, and the headwaters of the Pasquotank River. Since the mid-1980s, weirs have been installed throughout the study area. These structures have been particularly effective at raising water tables at the intersection of Corapeake and Laurel ditches (installed 1985), the intersection of Forestline and Cross Canal ditches (installed 1985), the intersection of South and Riddick ditches (installed in the 1990s), and a rock weir on Western Boundary Ditch (installed in the 1990s)(Fig. 1).

## Methods

Sampling of 11 stands was concurrent with salvage logging following Hurricane Isabel in September 2003. In each of these stands, 5–10 *C. thyoides* trees were randomly selected, and stem cross-sections were removed corresponding to breast height (1.4 m). Total ring-width subsamples were measured in four or five lines radiating from the center of each cross section. Ring widths from each measurement series were recorded to the nearest 0.001 mm using a Velmex TA micrometer system mounted above each cross section and Measure J2X software (Copenheaver et al. 2007). Series were then averaged as described by Patterson (2011) to create one total ring-width series for each stem cut following MacDonald and Yin (1999).

In two additional stands where stem cuts were not available, cores were extracted at breast height using a 4.3 mm-diameter increment borer in the summer of 2016. Where Heart Rot, an infection associated with a Polypore fungus which was described in *C. thyoides* by Korstian and Brush (1931), was evidenced by fragmentation within cores, coring was conducted higher up the stem or another tree was selected. When samples lacked pith, usually due to Heart Rot, the arc of the innermost ring of the tree was measured using a digital caliper to quantify the missing area and to estimate remaining radii. Borers were dipped in Garden Safe, Fungicide 3 between sampled trees. Samples were allowed to dry in the lab and were sanded with progressively finer grits of sandpaper. Ring width measurements were performed as described for stem cuts. Cores from all trees were visually cross-dated and dating accuracy was verified for stem cuts (11 stands) and cored (2 stands) samples using the program COFECHA (Holmes 1982; Cook 1985; Pederson et al. 2004).

*Chamaecyparis thyoides* radial growth was evaluated by creating basal area increment (BAI) chronologies from the total ring-width measurement series for each tree (Jenkins and Pallardy 1995). BAI reduces age-related trends in tree growth without eliminating growth responses resulting from other factors (Phipps and Whiton 1988) and has been used to assess growth and decline in trees (Livingston et al. 2017). The BAI for each year in each tree (BAI_t_) was calculated using the following formula:

$$BAI_{t} \, = \,\pi R_{t}^{2} - \pi R_{t - 1}^{2}$$

where R is the radius for year_t_ (Phipps and Whiton 1988). The BAI series for each tree were averaged to produce a BAI chronology for each stand in the study. BAI chronologies were evaluated for 1938–2002, the period common to all stands.

**Fig. 2:**
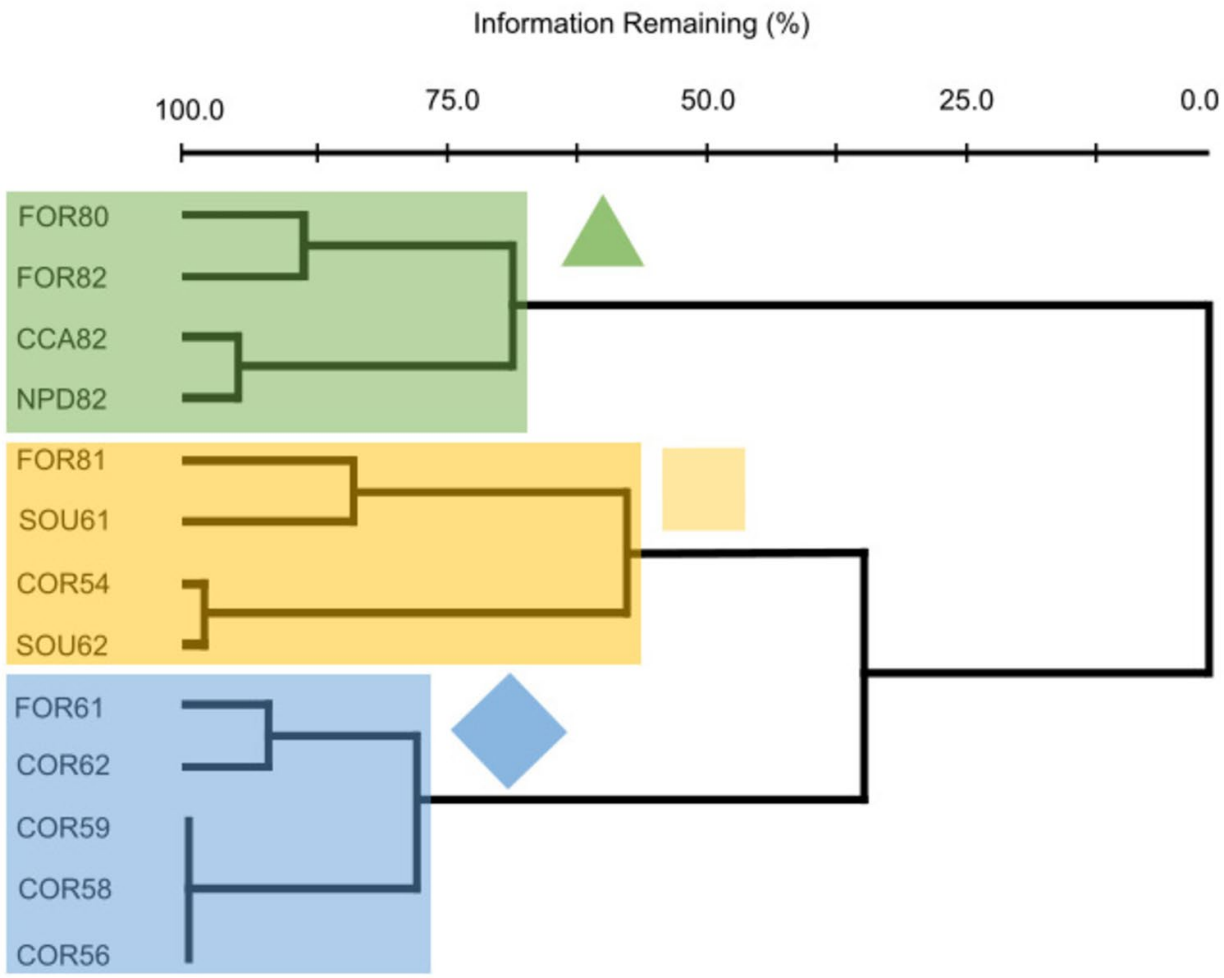
Cluster dendrogram of 13 study sites. Flexible beta cluster analysis yielded 3 groups: Corapeake/Laurel (green triangle, 4 stands totaling 32 trees as of 1929); Forestline/South/Riddick (yellow square, 4 stands totaling 85 trees as of 1964); and Forestline/South (blue diamond, 5 stands totaling 68 trees as of 1972). Numeric values in stand names represent mean age of trees at the end of the chronology

Radial growth patterns among stands were examined using flexible beta cluster analysis (beta = − 0.25) of a Sorensen distance matrix containing the BAI chronologies for each stand (Fig. 2). Flexible beta cluster analysis is a hierarchical, space-conserving technique similar to Ward’s Method (Lance and Williams 1967), but compatible with the Sorensen distance matrix. Groups were delineated based on natural breaks in the cluster dendrogram. Multi-response permutation procedures (MRPP) (Mielke 1984; Mielke and Berry 2001) were used to assess significant differences in hydrological characteristics among groups of stands with similar BAI patterns. We used partial mantel tests (Smouse et al. 1986) to directly examine the relationship between BAI chronologies for stands and environmental factors while controlling for stand age and stand location. Mantel tests examine the correlation between distance matrices and avoid the problem of dependence within each matrix (McCune and Grace 2002). To maintain consistency, a Sorensen distance matrix was used for the BAI chronologies, while environmental variables were examined using Euclidean distance matrices. Environmental variables in each matrix were transformed via relativization by maximum (McCune and Grace 2002). PC-ORD 7 software was used for all multivariate analyses (McCune and Mefford 2016).

Temporal variability in the mean of the BAI chronologies was examined among stand groups using regime shift analysis (RSA) (STARS 6.3; Rodinov 2004; Rodinov 2005a; Rodinov 2005b). RSA detects significant changes or shifts in the mean of adjacent regimes using a sequential application of the t-test (*P* < 0.05; cutoff length = 10 years). Shift detection was applied after the chronologies were pre-whitened to remove autocorrelation. A Huber weight function was used to reduce the potential effects of outliers on regime means. By examining shifts in the mean, we determined when a system changed from one hydrologic state or regime to another.

For each stand, we identified eight hydrologic variables to explain general hydrologic conditions at each stand:

- Distance to primary ditch—mapped straight-line distance (m) from the site to the ditch that has the most influence on hydrologic conditions at the stand.
- Distance to secondary ditch—mapped straight-line distance (m) from the stand to the closest ditch, after the primary ditch, to the stand.
- Presence or absence of a tertiary weir—mapped connection to a third weir that could influence hydrologic conditions at a stand based on existing flow patterns through the ditch network. The presence of a third weir is a reflection of the perceived complexity of the network of ditch and water control infrastructure that may influence hydrologic conditions at the stand.
- Stand age (years)—mean first year of tree width measurements through end of the chronology (2003) based on all trees in each stand.
- East/west slope (degrees)—mapped slope measured from the easternmost edge to westernmost edge of the stands using GoogleEarth (accessed July 2023).
- North/south slope (degrees)—mapped slope measured from the northernmost edge to the southernmost edge of the stands using GoogleEarth (accessed July 2023).
- Water table fluctuation—refuge personnel qualitative assessment of stands ranked (1 to 13) by estimated annual fluctuation in water table relative to the land surface at the stand.
- Duration of inundation—refuge personnel qualitative assessment of stands ranked (1 to 13) by estimated duration of inundation during the growing season.

## Results

Flexible beta cluster analysis of the 13 stands yielded 3 groups. Stand groups were named on the basis of the nearest ditch fitted with a weir, aka primary weir, and included Corapeake/Laurel (*n* = 5, triangle); Forestline/South (*n* = 4, diamond); and Forestline/South/Riddick (*n* = 4, square) (Fig. 2).

Corapeake/Laurel stands occurred on the flattest sites and contained the youngest *C. thyoides* trees. These stands were located in close proximity to primary and secondary ditches, and contained a rock weir which acted as a tertiary weir. Following weir installation, these stands exhibited the greatest fluctuation and duration in site hydroperiod.

Forestline/South stands occurred at higher elevation sites with steeper north–south slopes and contained the oldest *C. thyoides* trees. These stands were located at the greatest distances from primary and secondary ditches and did not contain a tertiary weir. Following weir installation, the least fluctuation and duration in site hydroperiod were observed within the Forestline/South stands.

Forestline/South/Riddick stands occurred on sites with the lowest elevations and steepest east–west slopes. These sites were located closest to a primary ditch, but relatively far from a secondary ditch. Half of the sites contained a rock weir acting as a tertiary weir. Following weir installation, fluctuation and duration in site hydroperiod were both low within Forestline/South/Riddick stands.

**Fig. 3:**
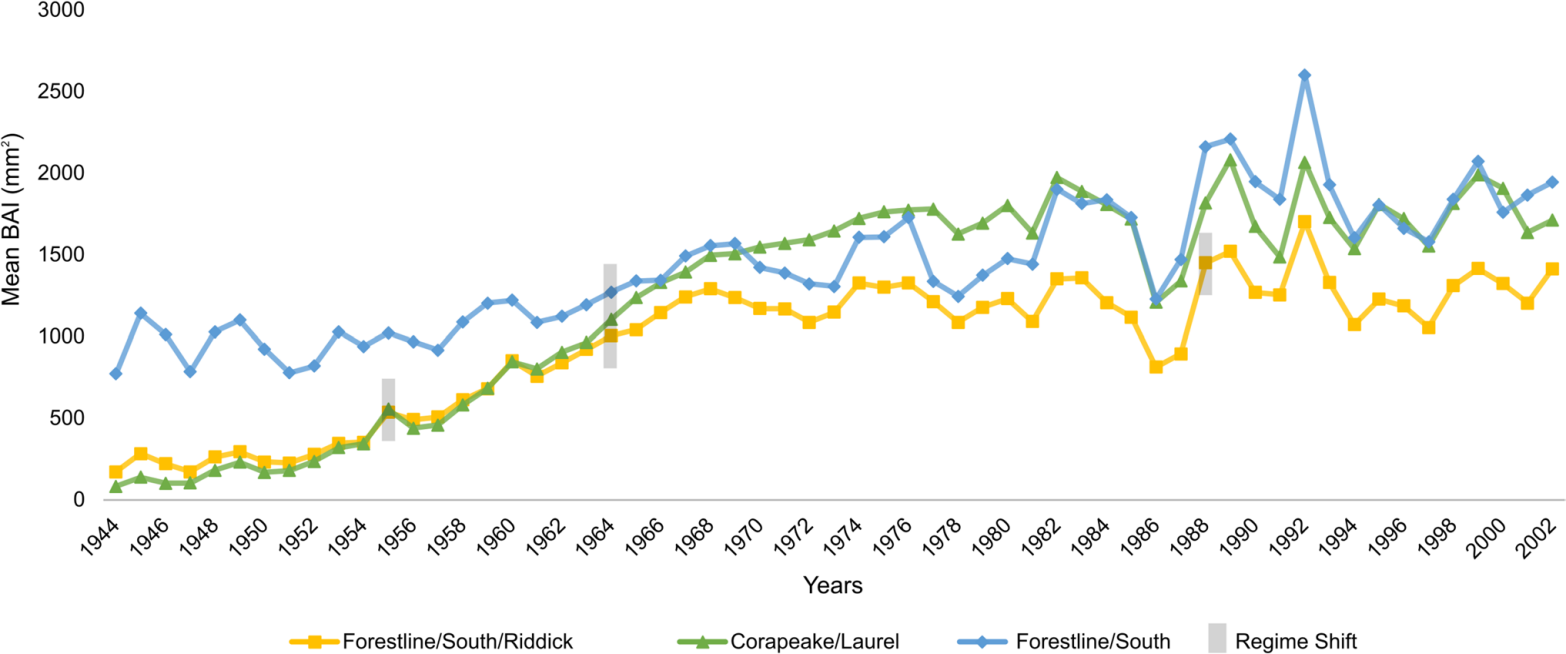
Mean basal area increment (BAI) in mm^2^ for the three groups: Corapeake/Laurel (green triangle); Forestline/South /Riddick (yellow square); and Forestline/South (blue diamond). Years of identified regime shifts are identified with grey boxing. The absolute sum RSI values (Regime Shift Index across sites) were 2.08 for 1955; RSI = 2.73 for 1964; and RSI = 0.47 for 1988. RSI is a cumulative measure of the exceedances beyond the prior regime’s significance value. A RSI > 0 is significant

According to multiple response permutation procedures (MRPP), environmental factors that differed significantly among stand groups were based on GDSNWR personnel qualitative assessment of hydroperiod in adjacent ditches, including both fluctuation (*P* = 0.03) and duration (*P* = 0.03); and mapped distances to primary ditches (*P* = 0.03) and to primary and tertiary weirs (*P* < 0.04)(Table 1). Stand age also differed among groups (*P* = 0.001). When age and location were held constant, partial mantel tests detected fewer significant environmental factors, but included mapped distances to primary ditches (*r* = 0.39, *P* = 0.04) and refuge personnel-assessed fluctuation in stand hydroperiod (*r* = 0.26, *P* = 0.03). Regime shifts in the mean BAI of each stand group for the common period (1939–2003) were identified for 2 years per group and generally confirm and expand Refuge personnel knowledge of periods of ditch construction and stand drainage history (Fig. 3). The Forestline/South and Corapeake/Laurel stand groups experienced regime shifts in mean BAI in 1955 and 1964, while the Forestline/South/Riddick group experienced shifts in mean BAI in 1964 and 1988 (Fig. 3).

**Table 1:** Multiple response permutation procedure tests of environmental matrices among *C. thyoides* stand groups

Environmental matrix	Forestline/South/Riddick^a*^	Corapeake/Laurel^b*^	Forestline/South^c*^	*T*^*1*^	*P*	*Delta*^*2*^	*A*^*3*^
Distance to primary ditch (m)	481.19^c^	180.78^c^	177.03^ab^	− 2.22	< 0.05	271.77	0.20
Distance to secondary ditch (m)	1158.73	233.35	675.92	− 0.37	0.30	688.30	0.03
Distance from primary weir to nearest point on ditch (m)	3921.70^b^	2454.25^ac^	2455.86^b^	− 2.17	< 0.05	2906.39	0.24
Distance from secondary weir to nearest point on ditch (m)	718.84	3462.77	1139.42	0.88	0.82	1724.89	− 0.06
Tertiary weir (presence or absence)	0.00^b^*	0.67^ac^	0.00^b^	− 3.36	< 0.01	0.21	0.62
Stand age (years)	0.90^c^	13.74^c^	2.74^ab^	− 3.76	< 0.01	5.56	0.56
East–West slope (degrees)	1.52	1.13	0.94	0.92	0.84	1.18	− 0.11
North–south slope (degrees)	1.45	0.88	0.84	0.30	0.54	1.04	− 0.03
Elevation (m)	3.83	0.67	2.20	0.74	0.75	0.74	− 0.07
Hydrological fluctuation via recent observations (rank)	2.17^c^*	5.17^c^	3.20^ab^	− 2.36	< 0.05	3.49	0.25
Hydrological duration via recent observations (rank)	2.17^c^*	5.17^c^	3.20^ab^	− 2.36	< 0.05	3.49	0.25

Mean Euclidean distances are presented for each stand group. Superscript letters, a–d, correspond to the order of the columns and summarize pairwise comparisons at *P* < 0.05 and *P < 0.10

^*^Higher Euclidean distances indicate greater heterogeneity within a group

*T*^*1*^ statistic and probability associated with the MRPP for each environmental matrix

*Delta*^*2*^ is the weighted mean within-group distance

*A*^*3*^ is the chance-corrected within-group agreement

## Discussion

### Hydrologic classification of stands

A temporarily flooded hydrologic regime is suggested for the entire study area and throughout the study duration, 1919 to 2003, based on BAI of individual stands and stand groupings. All 13 stands exhibited relatively high BAI growth rates (average 1093 mm^2^, Fig. 3), which were much faster than rates reported in a 60-year-old undrained reference stand (average BAI 240 mm^2^) which was characterized as having a seasonally flooded, saturated hydrologic regime (Atkinson 2020). The reference stand is 100 km southeast of GDSNWR in Alligator River National Wildlife Refuge (ARNWR) in northeastern North Carolina and exhibits similar hydrographs and growth rates to other undrained *C. thyoides* swamps in ARNWR (Doyle et al. 2021).

The cluster analysis placed the 13 stands into 3 stand groups (Fig. 2) and regime shifts occurred on 2 dates including 1954 for 2 groups (Corapeake/Laurel and Forestline/South) and 1964 for all 3 groups (Fig. 3). The shifts occur within a known ditching period (after 1953 and before 1970, Fig. 3) and provide new insights for dates of ditch installation or reactivation. The dendrogram sorted most of the 80-year-old stands (mean start year was 1919) into the Forestline/South group. Mean BAI for this group averaged ~ 1000 mm/year by the start of the common-age period, (1939–2003) (Fig. 3), a growth rate that also suggests a temporarily flooded hydrologic regime. The altered hydrology in this group may date to the establishment of a ditch, Cross Canal, which was established around 1888 (Shaler 1890). Globally, growth stimulation following stand drainage for silviculture has been widely recognized and drainage has been practiced in Fennoscandia since late in the last century (Trettin et al. 1997). Even brief periods of drawdown reverse peat accumulation and increase the risk of fire in southeast Asia forested peatlands (Dommain et al. 2010).

In addition to the regime shift analyses, ditch-related water level influences were also detected for 5 environmental variables using MRPP (Table 1); and, when age and latitude were held constant, partial mantel tests confirmed 2 of the 5 variables by including distance to a primary ditch (*P* = 0.033) and observed water level fluctuation in ditches (*P* = 0.057). When ditches are installed, water levels are lowered, particularly in close proximity to the ditches, resulting in rapidly increased bulk density concurrently with loss of ground elevation. Peat loss during combustion by fire or via microbial oxidation is as a result of peat drainage, either by ditching or by climate change, and has been described for peatlands in boreal regions (Beaulne et al. 2021), southeast Asia (Dommain et al. 2010; Page et al. 2009), and for Amazonian peatlands (Lahteenoja et al. 2009).

GDSNWR was formed in 1973 and refuge-forming documents called for prioritizing hydrologic restoration. A period of hydrologic restoration primarily via weir installations occurred between 1985 and 1995, and regime shift analysis detected a shift for 1 group (Forestline/South/Riddick) in 1986. In fact, growth rates for all 3 groups decreased by ~ 30% in 1986, but growth rates for all 3 groups more than recovered in just two years (1988), which indicates that hydrologic restoration was not fully achieved, further supports designation of a Temporarily Flooded hydrologic regime to the end of the chronology (2003), and suggests that water levels needed to be higher as of 2003.

Throughout the chronology, the highest growth rate variability, i.e., greatest climatic sensitivity, occurred after 1985 which is post-restoration (Fig. 3). Sensitivity to moisture-related variables such as Palmer Drought Severity Index (PDSI) was assessed by Atkinson (2020) for a stand within the Forestline/South/Riddick group of the current study. That study reported a positive correlation for PDSI and growth among 4 months during the growing season. Also working in that stand, Rodgers et al. (2003) reported fine root mortality both when water tables were higher in spring and again during late summer, presumably via desiccation. In contrast, in undrained stands such as the reference site and other undrained stands in the ARNWR, *C. thyoides* growth rate is somewhat complacent (Atkinson 2020; Doyle et al. 2021). In sum, the primary effect of drainage for trees in mid-Atlantic forested peatlands is growth enhancement as reported for boreal peatland conifers in several papers within Trettin et al. (1997). In addition, *C. thyoides* tree ring growth in drained sites also expresses greater climate sensitivity. Our 13 stands exhibit both of these indicators of temporarily flooded hydrologic regimes.

### Implications of a temporarily flooded hydrologic regime: peat loss by chronic microbial oxidation and acute fire

For at least 60 years, tree ring growth rate changes were detected by regime shift analyses, and rates were responsive to both proximity to ditches and to weirs, according to MRPP and partial mantel tests. Long-term drainage provides aerobic conditions that increase *C. thyoides* growth rates; but degrades peat conservation. Peat aeration from ditching facilitates peat-burning fires directly (Stricker et al. 2019), and indirectly by altered hydrophysical characteristics. Drained peat shrinks and forms fissures which create preferential surface flow (Schouwenaars 1988) which occurs in the upper 0.30–1.0 m in GDS allowing lateral flow to ditches (Speiran and Wurster 2021; Word et al. 2022; Link et al. 2023). Beneath this layer, most peatlands (Clymo 1984) and GDS (Speiran and Wurster 2021; Word et al. 2022) contain a near-permanently saturated layer with very weak hydraulic transmissivity. Working in proximity to the two stand groups that exhibited deep peat burns 9 years later, Atkinson et al. (2003) reported that GDSNWR water levels fell to 60 cm below the surface in just 30 days following a very wet period in early summer (1999). In contrast to the continuous saturation at ARNWR reference site, ditching in GDSNWR drained the upper layer of peat and established a temporarily flooded hydrologic regime which burned to a combined depth of 0.63 m during months long fires in 2008 and 2011 (Reddy et al. 2015; Hawbaker et al. 2016; Drexler et al. 2017; Sleeter et al. 2017), as predicted by *C. thyoides* growth patterns. A similar hydrologic sensitivity is known for *P. mariana*, a dominant tree in northern peatlands. As reported here for *C. thyoides*, stems of that conifer grow more slowly in seasonally flooded, saturated hydrologic regimes (Krause and Lemay 2023).

Temporarily flooded hydrologic regimes in *C. thyoides* stands also create conditions that chronically emit carbon via microbial respiration, as reported in GDSNWR using microcosms (Duttry et al. 2003) and field installations at GDSNWR (Kalnins 2000). Surface peats are no longer protected by anoxic conditions where water tables are lowered, and peat desiccation results in hydrophobic surfaces which severely alter capillary rise, from as much as 60 cm in undrained Scandinavian peats (Verry 1997). Capillary rise may have reached a threshold for lowered water levels (40 cm), which was described for forested peatlands in southeast Asia (Page et al. 2009), in spite of divergent peatland formation processes among global regions described by Dommain et al. (2010). Furthermore, hydrophobic surfaces form in some long drained forested peatlands in the mid-Atlantic region, and capillary rise of near 0 cm has been reported (Valat et al. 1991; Michel et al. 2001; Schimelpfenig et al. 1988). Working in long drained soils with histic epipedons near our stands, Johnson et al. (2022) reported sustained aerobic microbial respiration for weeks after soil microcosms were inundated.

### Implications associated with a temporarily flooded hydrologic regime: *C. thyoides* restoration

Seasonally flooded, saturated hydrologic regimes exhibit minimal fluctuation in depth to water table in *C. thyoides* swamps as a result of landscape position (Brinson 1993) leading to *Sphagnum* dominance and peat formation. Hydrologic modulation was eliminated during the long history of ditching in the Great Dismal Swamp which dewatered peat, and by the time Hurricane Isabel struck in 2003, only a 1200-ha (3000-acre) remnant remained. Changes in peat include formation of hydrophobic surfaces which reduce capillary rise (which may be irreversible as described by Dolman and Buol (1967)), and peat shrinkage which can facilitate lateral flow (Atkinson et al. 2003). These hydrophysical conditions allow water level fluctuation that is tolerated by trees in other forested wetland types (Megonigal et al. 1997) and some boreal peatland tree species (Krause and Lemay 2023), but are lethal to *C. thyoides*. Several studies report high mortality rates among inundated *C. thyoides* propagules including natural regenerants, seedlings, and rooted cuttings (Harrison et al. 2003; Cook et al. 2015; Foster et al. 2015; Wurst et al. 2015). When inundated, *C. thyoides* produces ethanol which can allow air flow from aboveground organs to roots (Kelsey et al. 2011), but this anatomy could increase drought stress if water levels fall beneath the root zone (Kozlowski 1979; Kozlowski and Pallardy 1997), as reported for naturally regenerating and planted *C. thyoides* in GDS (Cook et al. 2015).

Reestablishment of *Sphagnum* may be required prior to *C. thyoides* reestablishment, but may be difficult to achieve by rewatering peat (Schouwenaars 1988; Smolders et al. 2003). *Chamaecyparis thyoides* reestablishment efforts in GDS may require a higher water level than that tolerated by mesophytic species which have displaced *C. thyoides* including *Acer rubrum* and *Nyssa sylvatica* Marshall. Once *Sphagnum* is reestablished, *C. thyoides* plantings may be more successful on mounds than in pools, or atop beds where the silvicultural practice of bedding is practical.

Many tree species exhibit radial growth rates that respond to drought in a manner that is influenced by hydrologic regime, e.g., Beech, *Fagus grandifolia* Ehrh. (Copenheaver et al. 2007; Dudek et al. 1998); however, *C. thyoides* is uniquely suited for assessing hydrologic conditions in mid-Atlantic peatlands such as the Dismal Swamp. *Pinus taeda* has been proposed to indicate hydrologic regimes in the Dismal Swamp (Phipps et al. 1978); however, as a facultative hydrophyte, occurrence is infrequent in local swamps having a seasonally flooded, saturated hydrologic regime (Shacochis et al. 2003). *Taxodium distichum* (L.)Rich. occurs across a range of hydrologic regimes but may be too tolerant of long-term soil anoxia to exhibit growth limitations associated with saturation (Allen and Keim 2017) and responses to inundation may be slow to develop (Young et al. 1995). The dominance of *A. rubrum* has increased greatly as mid-Atlantic peatlands were drained (Laderman 1989), especially in Dismal Swamp (Levy and Walker 1979; Ludwig et al. 2021) and this species exhibits broad tolerance of hydrologic regimes. However, Seim et al. (2006) reported that radial growth sensitivity of *C. thyoides* was greater than for *A. rubrum* for both current year temperature and precipitation and that false and missing rings are common in *A. rubrum*.

Mid-Atlantic *C. thyoides* peatlands form in a unique hydrogeomorphic setting which favors paludification, establishment of hydrophilic peat and dominance by *Sphagnum*, forming a persistently saturated hydrologic regime, termed seasonally flooded, saturated. These conditions favor dense and slow growing stands *C. thyoides* which supports critical ecosystem services including carbon sequestration as well as biodiversity support, among others. As of 2003, *C. thyoides* stands exhibited a similar growth signature among all 13 stands allowing classification of a temporarily flooded hydrologic regime classification within a large portion of the GDSNWR. While saturated conditions become anoxic and greatly reduce *C. thyoides* annual growth rates, MRPP and partial mantel tests found that GDS ditching was so extensive that drained sites in GDS grew slower than the less drained sites and Atkinson (2020) found a positive response to precipitation typical of drier sites. Following refuge formation and installation of weirs until 2003, tree ring analysis via MRPP detected a reduced growth response that was still somewhat faster than growth of the reference site. And in 2001, a 4-year study of *C. thyoides* ecology omitted dendroecological indicators yet concluded that water levels in GDSNWR were “not adequate to curtail microbial decomposition of organic matter and the risk of long-term damage due to fire is high (high probability and high severity)” (Atkinson et al. 2001). The insufficient hydrologic restoration achieved through 2003 may have contributed to the 2008 and 2011 fires burning more than 1000 years of accumulated peat (Hawbaker et al. 2016) and emitting 1.70 Tg of carbon, 1.38 Tg of which were peat (Sleeter et al. 2017).

## Conclusion

Globally, peatlands form where persistent soil saturation reduces peat oxidation more than it restricts primary production leading to a positive carbon budget and carbon storage in soils. Those anoxic soil conditions increase stress on some forested peatland tree species such as *C. thyoides* and decrease growth as recorded in tree rings. In this study, historical knowledge of the timing of human activities (ditch installation and deepening, and installation of weirs) was confirmed and refined by tree ring growth patterns. The wider rings in *C. thyoides* indicated lower water tables and increased peat loss through chronic increases in microbial oxidation and acute soil carbon oxidation via catastrophic fire.

Additional insights into peatland hydrologic regimes and hydrologic restoration were presented in our study. The capillary rise in undrained, hydrophilic peat soils helps maintain soil saturation as much as 60 cm above the water table in forested boreal peatlands, but long-term drainage reverses this function. In addition, while tropical peatland hydrology is not influenced by *Sphagnum*, these mosses are known to support peat formation in boreal and temperate regions. Peat hydrophobicity is not reversible; therefore, it appears likely that peat restoration and revegetation by *C. thyoides* will require establishment of *Sphagnum*.

Tree ring widths for all 13 stands in this study suggest an extensive period of anthropogenic drainage resulting in lower water tables and hydrophobic peat surfaces that support little to no capillary rise. The result has intensified peat loss via both chronic (microbial oxidation) and acute (soil-burning) fire which consumed at least 1,000 years of accumulated peat.

## Data Availability

Data are available through the corresponding author.
